# Applicability of *HIN-1, MGMT* and *RASSF1A* promoter methylation as biomarkers for detecting field cancerization in breast cancer

**DOI:** 10.1186/s13058-015-0637-5

**Published:** 2015-09-14

**Authors:** Melanie Spitzwieser, Elisabeth Holzweber, Georg Pfeiler, Stefan Hacker, Margit Cichna-Markl

**Affiliations:** Department of Analytical Chemistry, University of Vienna, Währinger Str. 38, 1090 Vienna, Austria; Department of Obstetrics and Gynecology, Division of Gynecology and Gynecological Oncology, Medical University of Vienna, Währinger Gürtel 18-20, 1090 Vienna, Austria; Department of Plastic and Reconstructive Surgery, Medical University of Vienna, Währinger Gürtel 18-20, 1090 Vienna, Austria

## Abstract

**Introduction:**

It has been shown in some articles that genetic and epigenetic abnormalities cannot only be found in tumor tissues but also in adjacent regions that appear histologically normal. This phenomenon is metaphorically called field cancerization or field defect. Field cancerization is regarded as clinically significant because it is assumed to be an important factor in local recurrence of cancer. As the field showing these molecular abnormalities may not be removed completely by surgery, these changes might lead to neoplasms and subsequent transformation to a tumor. We aimed to investigate the applicability of the methylation status of six tumor suppressor genes as biomarkers for detecting field cancerization in breast cancer.

**Methods:**

The promoter methylation status of *CCND2*, *DAPK1*, *GSTP1*, *HIN-1*, *MGMT* and *RASSF1A* was determined by methylation-sensitive high-resolution melting (MS-HRM) analysis. MS-HRM methods for *CCND2*, *MGMT* and *RASSF1A* were developed in-house, primer sequences for *DAPK1*, *GSTP1* and *HIN-1* have already been published. Biopsy samples were taken from tumor, tumor-adjacent and tumor-distant tissue from 17 breast cancer patients. Normal breast tissues of four healthy women served as controls.

**Results:**

All MS-HRM methods proved to be very sensitive. LODs were in the range from 0.1 to 1.5 %, LOQs ranged from 0.3 to 5.3 %. A total of 94 %, 82 % and 65 % of the tumors showed methylation of *RASSF1A*, *HIN-1* an*d MGMT* promoters, respectively*.* The methylation status of these promoters was significantly lower in tumor-distant tissues than in tumor tissues. Tumor-adjacent tissues showed higher methylation status of *RASSF1A*, *HIN-1* and *MGMT* promoters than tumor-distant tissues, indicating field cancerization. The methylation status of the *HIN-1* promoter in tumor-adjacent tissues was found to correlate strongly with that in the corresponding tumors (r = 0.785, *p* < 0.001), but not with that in the corresponding tumor-distant tissues (r = 0.312, *p* = 0.239).

**Conclusions:**

Among the gene promoters investigated, the methylation status of the *HIN-1* promoter can be considered the best suitable biomarker for detecting field cancerization. Further investigation is needed to test whether it can be used for defining surgical margins in order to prevent future recurrence of breast cancer.

## Introduction

Cancer can be considered as cumulative phenotypic consequence of acquired genetic and epigenetic alterations in cells [1]. Epigenetic alterations, in particularly changes in the DNA methylation pattern, are known to play a crucial role in carcinogenesis. Aberrant DNA methylation occurs early in carcinogenesis, suggesting that DNA methylation alterations may precede classical transforming events such as gene mutations. Changes in the DNA methylation status occur more frequently than mutations or cytogenic abnormalities [2].

In humans and other mammals, DNA methylation takes place at carbon-5 of cytosine residues within cytosine-phosphatidyl-guanosine (CpG) dinucleotides. CpG dinucleotides are heterogeneously distributed in the human genome, often clustered in so-called CpG islands. CpG islands are particularly present in promoter regions and first exons of genes that regulate important cell functions [3].

In normal cells, CpG islands are generally unmethylated, resulting in gene expression, if the corresponding transcription factors are available [2]. In cancer cells, however, the promoter region of certain genes is frequently hypermethylated, leading to a tightly packed chromatin and transcriptional gene silencing. Promoter hypermethylation commonly affects regulator genes that are involved in a wide range of cellular pathways, such as cell cycle, DNA repair, toxic catabolism, cell adherence, apoptosis and angiogenesis [4].

Several studies have already investigated the applicability of promoter hypermethylation as specific and sensitive biomarkers, e.g., for the detection and diagnosis of cancer at an early stage [5, 6], the prognosis of cancer [7, 8] or the prediction of the response to a certain treatment scheme [9, 10]. In contrast to genetic alterations, changes in the DNA methylation status are potentially reversible. Reactivation of epigenetically silenced genes by using DNA demethylating drugs is therefore regarded as a promising strategy in cancer therapy [11, 12].

Recent studies have shown that molecular abnormalities occur not only in the tumor tissue but also in tissue that is adjacent to the tumor and appears histologically normal. The presence of such abnormalities in tissues surrounding tumors is called field cancerization or field defect [13]. In addition to genetic abnormalities, e.g., chromosomal anomalies and loss of heterozygosity, epigenetic alterations, in particular changes in the DNA methylation status, have been found in normal-appearing tissues close to tumors. Molecular signatures of field cancerization have been reported for various epithelial tumors including those of the colon [14–16] and the prostate [17, 18].

Field cancerization is of clinical relevance because it is assumed to be an important factor in local recurrence of cancer [19]. As the field showing aberrant DNA methylation may not be removed completely by surgery, these changes in the DNA methylation status might lead to neoplasms and subsequent transformation to a tumor. So far, only a limited number of studies have investigated changes in the DNA methylation status in histologically normal tissue adjacent to breast tumor tissues. Yan et al*.* [20] detected methylation changes in the promoter of Ras association domain family member 1 (*RASSF1A*) in mammary tissue as far as 4 cm from the primary tumor. In a study of Feng et al. [21] the DNA methylation status of reversion-induced LIM protein (*RIL*), high in normal-1 (*HIN-1*), *RASSF1A* and cadherin-13 (*CDH13*) in normal-appearing tissue (located at least 3 cm away from the tumor) was found to correlate with that in the breast tumor.

The main aim of the present study was to extend research on the applicability of the methylation status of candidate genes as biomarkers for field cancerization. We selected a panel of six tumor suppressor genes that have previously been reported to be frequently methylated in breast tumors, comprising cyclin D2 (*CCND2*), death-associated protein kinase 1 (*DAPK1*), glutathione S-transferase P1 (*GSTP1*), *HIN-1*, O6-methylguanine-DNA methyltransferase (*MGMT*) and *RASSF1A* [22]. From each of 17 breast cancer patients, three biopsy samples were taken: the first one from the tumor tissue, the second one from tumor-adjacent tissue and the third one from tumor-distant tissue. In addition, we tested if the DNA methylation status of the six tumor suppressor genes in tumor, tumor-adjacent and/or tumor-distant tissues is associated with any clinicopathological parameters. We were also interested to see if there is a correlation between the DNA methylation status of the genes in the tissues of one and the same breast cancer patient.

## Methods

### Patients and biopsy samples

The study was approved by the Ethics Commission of the Medical University of Vienna (application number 1074/2011). All patients gave written informed consent. Biopsy samples from 17 breast cancer patients (aged 39–76 years, mean age: 58 years) were taken by ultrasound-guided needle biopsies. None of the patients had a family history of breast cancer. From each patient, three biopsy samples were taken: the first one directly from the primary breast tumor, the second one from histologically normal tissue located about 1 cm from the tumor (“tumor-adjacent tissue”) and the third one from histologically normal tissue located about 3 cm away from the tumor (“tumor-distant tissue”). In addition, breast tissue samples were obtained from four women (aged 44–60 years, mean age: 53 years) undergoing breast reduction mammoplasty. From two of these women, samples were obtained from both the left and right breast.

All biopsy samples were stored in phosphate-buffered saline (PBS) at −80 °C until analysis.

### Patient characteristics

Patient characteristics including age, menopausal status, histologic type, histological grading, B classification, proliferative activity (MIB-1), status of estrogen receptor (ER), progesterone receptor (PR) and human epidermal growth factor receptor 2 (HER2) as well as the molecular subtype are summarized in Table 1. Information on menopausal status, histological grading, MIB-1 and the receptor status was, however, lacking for one, two, one and one patients, respectively.

**Table 1:** Characteristics of breast cancer patients

Patient	Age (y)	Menopause status	Histologic type	Histological grading	B classification	MIB-1 (%)	Receptor status			Molecular subtype
							ER	PR	HER2	
1	75	Post	IDC	2	5b	10	+	+	−	Luminal A
2	65	Post	IDC	2	5b	10	+	+	−	Luminal A
3	54	n.s.	IDC	3	5b	n.s.	n.s.	n.s.	n.s.	n.s.
4	39	Pre	IDC	2	5b	40	+	+	+	Luminal B
5	66	Post	IDC	2	5b	60	+	+	−	Luminal A
6	50	Pre	IDC	3	5b	50	+	+	+	Luminal B
7	73	Post	IDC	3	5b	20	+	+	−	Luminal A
8	76	Post	IDC	2	5b	20	+	+	−	Luminal A
9	63	Post	IDC	3	5	30	+	+	−	Luminal A
10	48	Post	IDC	3	5b	20	+	+	+	Luminal B
11	58	Post	IDC	n.s.	5c	20	+	+	+	Luminal B
12	61	Post	IDC	3	5b	70	−	−	−	Triple negative
13	52	Pre	ILC	n.s.	5b	50	+	+	−	Luminal A
14	42	Pre	IDC	3	5b	80	+	−	−	Luminal A
15	67	Post	IDC	3	5b	40	+	+	−	Luminal A
16	61	Post	ILC	2	5b	30	+	+	−	Luminal A
17	41	Pre	Mucinous	2	5b	50	+	+	+	Luminal B

MIB-1 mindbomb E3 ubiquitin protein ligase 1, *ER* estrogen receptor, *PR* progesterone receptor, *HER2* human epidermal growth factor receptor 2, IDC invasive ductal carcinoma, ILC invasive lobular carcinoma, n.s. not specified

### Extraction of genomic DNA

Genomic DNA was extracted from biopsy samples using the QIAamp DNA Mini Kit (Qiagen, Hilden, Germany) according to the manufacturer’s protocol. The DNA concentration was determined using a NanoDrop 2000c spectrophotometer (Thermo Fisher Scientific, Waltham, MA, USA).

### Methylation-sensitive high-resolution melting (MS-HRM) analysis

DNA extracted from biopsy samples and human control DNA (fully methylated and unmethylated) were treated with sodium bisulfite using the EpiTect Fast DNA Bisulfite Kit (Qiagen) according to the manufacturer’s instructions. Fully methylated control DNA (CpGenom Universal Methylated DNA) was obtained from EMD Millipore (Billerica, MA, USA), unmethylated control DNA (EpiTect Control DNA (human), unmethylated) from Qiagen.

Primers for *CCND2* (GenBank: CM000263.1), *MGMT* (GenBank: X61657.1) and *RASSF1A* (GenBank: NG_023270.1)*,* targeting CpG island regions flanking the transcription site, were designed with the Methyl Primer Express Software v1.0 (Applied Biosystems, Carlsbad, CA, USA). Primer sequences for *DAPK1* [23], *GSTP1* [24] and *HIN-1* [25] were taken from the literature. For each MS-HRM method, the annealing temperature (T_a_) and the MgCl_2_ concentration were optimized in-house. Primer sequences and optimized conditions are summarized in Table 2.

**Table 2:** Primer sequences and methylation-sensitive high-resolution melting (MS-HRM) conditions

Gene	Primer sequences	Additional MgCl_2_ concentration (mM)	T_a_ (°C)	Amplicon length (bp)	Number of CpGs	LOD / LOQ (%)	Reference
*CCND2*	F: 5′ GTTTTAGAGCGGAGAAGAG 3′	0	50	89	4	0.1 / 0.3	In-house
	R: 5′ AACAAAACCTCGAAACTACC 3′						
*DAPK1*	F: 5′ GCGCGGAGTTGGGAGGAG 3′	0	57	70	7	0.2 / 0.9	[23]
	R: 5′ CTCCGAACTACCCTACCAAACC 3′						
*GSTP1*	F: 5′ GTGAAGCGGGTGTGTAAGTTT 3′	1	56	120	12	0.9 / 3.3	[24]
	R: 5′ TAAACAAACAACAAAAAAAAAACC 3′						
*HIN-1*	F: 5′ GCGAGGATCGGGTATAAGAAGTT 3′	2	55	133	12	0.3 / 1.4	[25]
	R: 5′ CACCGAAACATACAAAACAAAACCA 3′						
*MGMT*	F: 5′ TTGATTAGGGGAGCGGTATTAG 3′	2	52	140	14	0.9 / 3.0	In-house
	R: 5′ CCACATACCCGAATAATCCTAAAA 3′						
*RASSF1A*	F: 5′ GTCGGGGTTTGTTTTGTGGTT 3′	2	56	118	9	1.5 / 5.3	In-house
	R: 5′ CAACTCCCACAACTCAATAAACT 3′						

*T*_*a*_ annealing temperature, *bp* base pair, *CpG* cytosine-phosphatidyl-guanosine, *LOD* limit of detection, *LOQ* limit of quantification

Polymerase chain reaction (PCR) amplification of the bisulfite-treated DNA and HRM analysis were performed using a Rotor-Gene Q thermocycler (Qiagen). Each reaction mixture had a total volume of 20 μl, containing 10 ng of bisulfite-treated DNA, 10 μl of 2× EpiTect HRM PCR Master Mix (Qiagen), forward and reverse primer and RNase-free water. In all PCR reactions, the concentration of forward and reverse primer was 250 nM each. PCR amplification was carried out under the following conditions: initial step at 95 °C for 5 min; followed by 50 cycles at 95 °C for 10 s, T_a_ of the respective primer set for 30 s and 72 °C for 10 s (touchdown 1 °C, 7 cycles); denaturation step at 95 °C for 1 min followed by a hybridization step at 40 °C for 1 min. In the HRM step, the temperature was increased by 0.1 °C increments per 2 s.

HRM data were evaluated with the Rotor-Gene Q Series Software 2.1.0 (Qiagen). Each biopsy sample was analyzed at least twice in duplicate. The DNA methylation status was calculated with the help of calibration curves established by analyzing mixtures of fully methylated and unmethylated human control DNA. In order to obtain accurate results also for heterogeneously methylated sequences, an interpolation calibration curve was established as proposed by Migheli et al*.* [26]. However, we slightly changed their approach and calculated the average of the normalized fluorescence signal for each standard over the entire temperature interval instead of using single values at chosen temperature points. Calibration functions were established with SigmaPlot 11.0 (Systat Software Inc., San Jose, CA, USA). Limit of detection (LOD) and limit of quantification (LOQ) of the MS-HRM methods were determined by repeatedly analyzing bisulfite-treated, unmethylated control DNA. After calculating the mean and the standard deviation, the LOD (signal-to-noise ratio (S/N) of 3) was determined by adding three times the standard deviation and the LOQ (S/N of 10) by adding ten times the standard deviation to the mean. The corresponding methylation status was then calculated with the help of the equation of the calibration curves established by analyzing mixtures of methylated and unmethylated control DNA.

### Statistical analysis

Statistical analyses were carried out with IBM SPSS Statistics 21.0 (IBM Corp., Armonk, NY, USA). The methylation status was treated either as categorical variable (< LOD, < LOQ or ≥ LOQ) or as continuous variable. If the methylation status was treated as continuous variable, methylation status < LOD and < LOQ were substituted with a default value, namely half the LOD and half the LOQ, respectively, as proposed previously [27]. Chi-squared test was used to evaluate if the methylation status of the six genes is associated with any of the clinicopathological parameters. Independent-samples *t* test was applied to evaluate if there are significant differences in the methylation status between the tumor tissues and noncancerous breast tissues of the control group. One-way ANOVA (analysis of variance), followed by Tukey’s test, was applied to test for significant differences in the DNA methylation status between tumor, tumor-adjacent and tumor-distant tissues. Levene’s test was used to assess the equality of variances. Pearson’s correlation coefficient was used to assess the relationship between two continuous variables. In all cases, *p* < 0.05 (two-sided) was considered significant.

## Results

### Validation of the MS-HRM methods

**Fig. 1:**
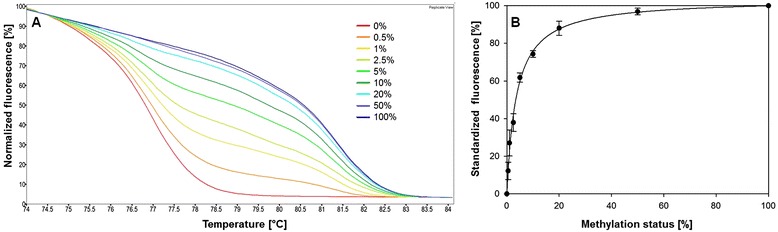
Repeatability of the high-resolution melting (HRM) method for *DAPK1*. **a** Normalized melting curves of mixtures of unmethylated and fully methylated control DNA. Methylation status of the standards: 0 %, 0.5 %, 1 %, 2.5 %, 5 %, 10 %, 20 % 50 %, 100 %. Replicate view of duplicate measurements carried out on 1 day. **b** Calibration curve obtained by analyzing mixtures of unmethylated and fully methylated control DNA in duplicate on 4 different days (n = 8)

MS-HRM methods for *CCND2*, *MGMT* and *RASSF1A* were developed in-house, primer sequences for *DAPK1*, *GSTP1* and *HIN-1* were taken from previously published articles. After optimizing the T_a_ and the MgCl_2_ concentration added to the commercial HRM PCR Master Mix (Table 2), the methods were validated with regard to limit of detection (LOD), limit of quantification (LOQ) and inter-day repeatability. LOD and LOQ of the MS-HRM methods were determined by repeatedly analyzing bisulfite-treated, unmethylated control DNA. LODs (S/N = 3) and LOQs (S/N = 10) were in the range from 0.1 to 1.5 % and 0.3 to 5.3 %, respectively (Table 2). These data demonstrate that the MS-HRM methods are applicable to detect and quantify low methylation levels. The repeatability of the methods was investigated by repeatedly analyzing mixtures of unmethylated and fully methylated control DNA. Figure 1a shows normalized melting curves of mixtures of unmethylated and fully methylated control DNA for *DAPK1*. The representative calibration curve, obtained by analyzing mixtures of unmethylated and fully methylated control DNA on 4 days in duplicate, indicates the high inter-day repeatability of the method (Fig. 1b).

### Heterogeneous methylation

**Fig. 2:**
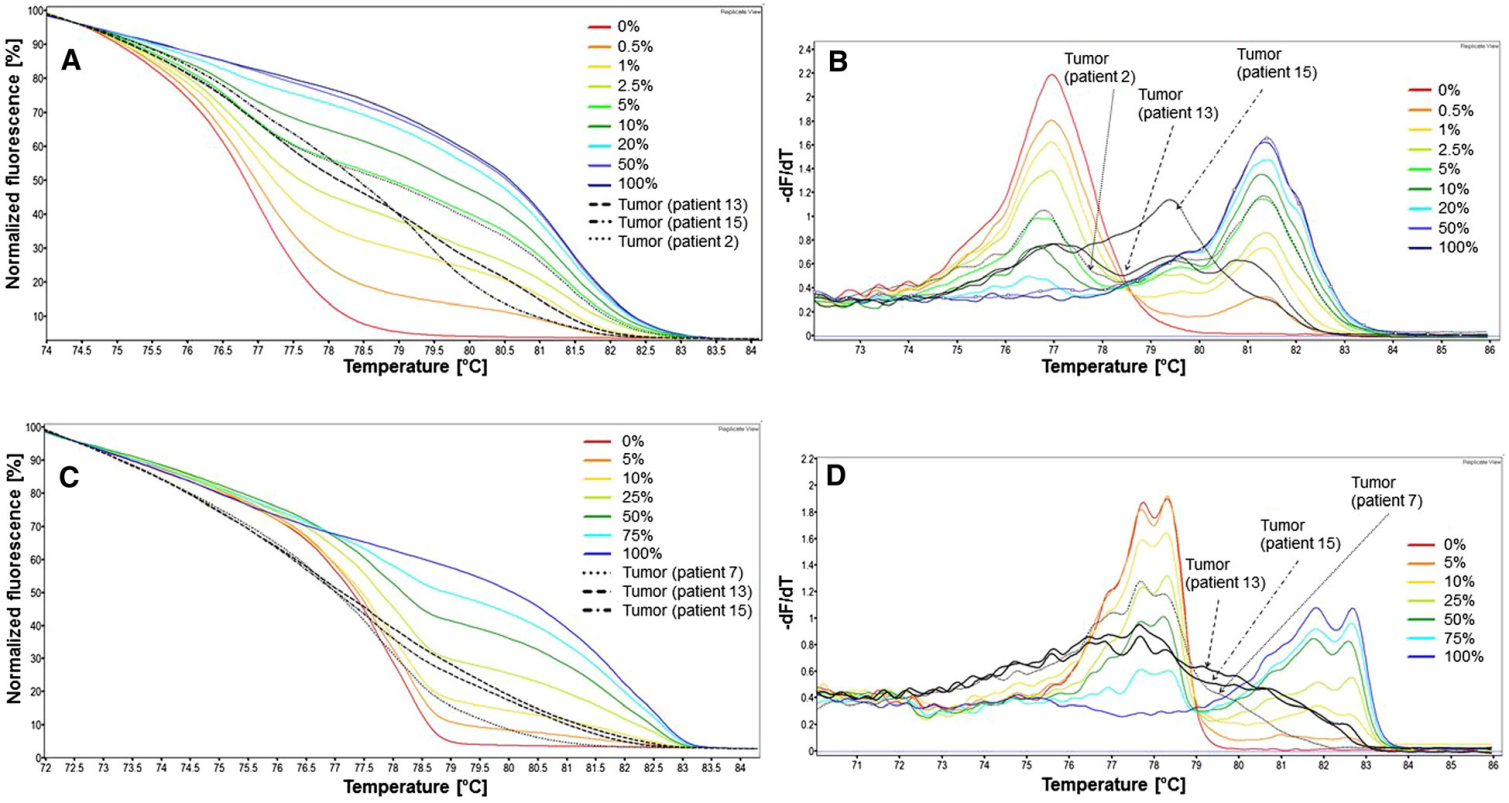
Heterogeneous methylation of the *DAPK1* and *MGMT* promoters in some tumor tissues. Normalized melting curves for *DAPK1* (**a**) and *MGMT* (**c**) and corresponding derivative plots (negative first derivative of the melting curves; **b** and **d**). Tumor tissue from patients 2, 13 and 15 (**a** and **b**) and tumor tissue from patients 7, 13 and 15 (**c** and **d**)

MS-HRM methods make it possible to determine the methylation status across all CpG dinucleotides within the amplicon, but they do not allow determining the methylation status of individual CpG dinucleotides. However, in contrast to several other methods, MS-HRM analysis is applicable to detect heterogeneous methylation [28–30]. Melting curves obtained for heterogeneously methylated templates differ in the shape from those obtained for mixtures of unmethylated and fully methylated control DNA. In addition, the melt profile derivative plots do not contain distinct peaks as observed for unmethylated and fully methylated DNA (Fig. 2). When we applied the MS-HRM methods to biopsy samples of breast cancer patients, the promoters of *CCND2*, *GSTP1*, *HIN-1* and *RASSF1A* were generally found to be methylated homogeneously. Melting profiles obtained for *MGMT* and *DAPK1* indicated that most biopsy samples showed homogeneous methylation whereas in some samples, *MGMT* and/or *DAPK1* were methylated heterogeneously (Fig. 2).

### Methylation status in breast tissues of healthy controls

DNA extracts from breast tissues of four healthy women that had been undergoing breast reduction mammoplasty were analyzed to determine DNA methylation base levels in noncancerous breast tissues. In all breast tissues of the control group, the methylation status of *DAPK1*, *HIN-1* and *RASSF1A* was < LOD. In three tissues, the methylation status of *CCND2*, *GSTP1* and *MGMT* was found to be < LOD. In one tissue, the methylation status of *GSTP1* was < LOQ. In the breast tissue of another woman, *CCND2* and *MGMT* were found to be methylated (*CCND2*: < LOQ, *MGMT*: 5.1 %). From two of the four women we obtained tissues from the left and right breast. In these women, no difference was found between the methylation status in the left and in the right breast. For none of the tumor suppressor genes did we find an association between the age of the women and the DNA methylation status.

### Methylation status in tumor, tumor-adjacent and tumor-distant tissue

**Fig. 3:**
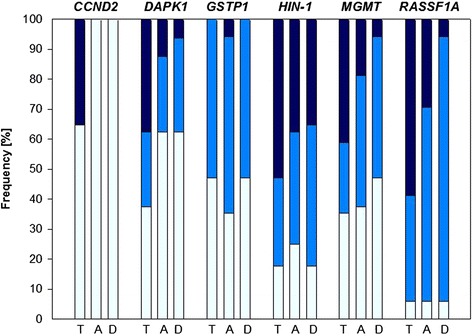
Frequency of promoter methylation in tumor (*T*), tumor-adjacent (*A*) and tumor-distant (*D*) tissues. *Light blue*: methylation status < LOD, *middle blue*: methylation status < LOQ, *dark blue*: methylation status ≥ LOQ. *LOD* limit of detection, *LOQ* limit of quantification

In spite of the low LOQs of the MS-HRM methods, a rather high proportion of the tumor tissues, tumor-adjacent tissues (located about 1 cm from the tumor) and tumor-distant tissues (located 3 cm from the tumor tissue) was found to show methylation status < LOQ. In several samples, the promoter was unmethylated (< LOD). Figure 3 shows the percentage of tumor, tumor-adjacent and tumor-distant tissues having methylation status < LOD, <LOQ and ≥ LOQ for each of the six tumor suppressor genes.

In tumor tissues, the promoters of five of the six genes were frequently methylated (methylation status ≥ LOD). A total of 94 % (16/17), 82 % (14/17), 65 % (11/17), 63 % (10/16) and 53 % (9/17) of the tumors showed promoter methylation of *RASSF1A*, *HIN-1*, *MGMT*, *DAPK1*, and *GSTP1*, respectively. The promoter of *CCND2* was methylated in only 35 % (6/17) of the tumors. In each of the 17 tumors, the promoter of at least one of the six genes was found to be methylated. Twelve tumors (71 %) showed promoter methylation of ≥ 4 genes. In 82 % (14/17) of the tumors, the promoters of both *RASSF1A* and *HIN-1* were found to be methylated. In general, the methylation status was, however, rather low. In only 59 % (10/17), 53 % (9/17), 41 % (7/17), 38 % (6/16) and 35 % (6/17) of the tumor samples, *RASSF1A*, *HIN-1*, *MGMT*, *DAPK1* and *CCND2* promoters showed methylation status ≥ LOQ. In none of the tumors, *GSTP1* promoter methylation was ≥ LOQ. In 41 % (7/17) of the tumors, both *RASSF1A* and *HIN-1* promoters showed methylation status ≥ LOQ.

In tumor-adjacent and tumor-distant tissues, the *RASSF1A* promoter was as frequently and the *HIN-1*, *MGMT* and *GSTP1* promoters were almost as frequently methylated as in tumors. In only 38 % (6/16) of the tumor-adjacent and tumor-distant tissues, the *DAPK1* promoter was found to be methylated, compared to 63 % (10/16) in tumors. *CCND2* was not methylated in any of the tumor-adjacent or tumor-distant tissues.

Figure 3 indicates that the percentage of tumor-distant tissues showing methylation status of *RASSF1A*, *MGMT* or *DAPK1* promoters ≥ LOQ was lower (6 %) than the percentage of tumor-adjacent tissues (29 % (5/17), 19 % (3/16) and 13 % (2/16), respectively), which, in turn, was lower than the percentage of tumor tissues (59 % (10/17), 41 % (7/17) and 38 % (6/16), respectively). With 35 % (6/17) and 38 % (6/16), the *HIN-1* promoter rather frequently showed methylation ≥ LOQ in tumor-adjacent and tumor-distant tissues, respectively. In contrast, in only one of the tumor-adjacent tissues and none of the tumor-distant tissues, the methylation status of the *GSTP1* promoter was ≥ LOQ.

**Fig. 4:**
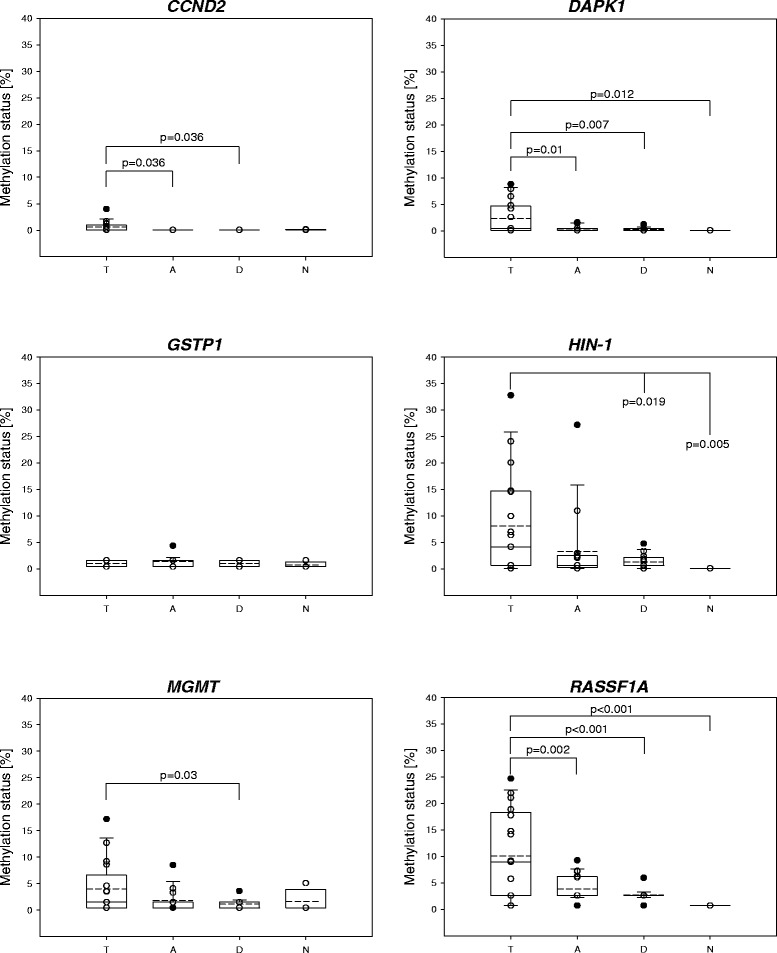
Distribution of the methylation status in tumor (*T*), tumor-adjacent (*A*) and tumor-distant (*D*) tissues as well as normal breast tissues obtained from women undergoing reduction mammoplasty (*N*). All patients have been included. *Straight line*: median, *dashed line*: arithmetic mean

**Fig. 5:**
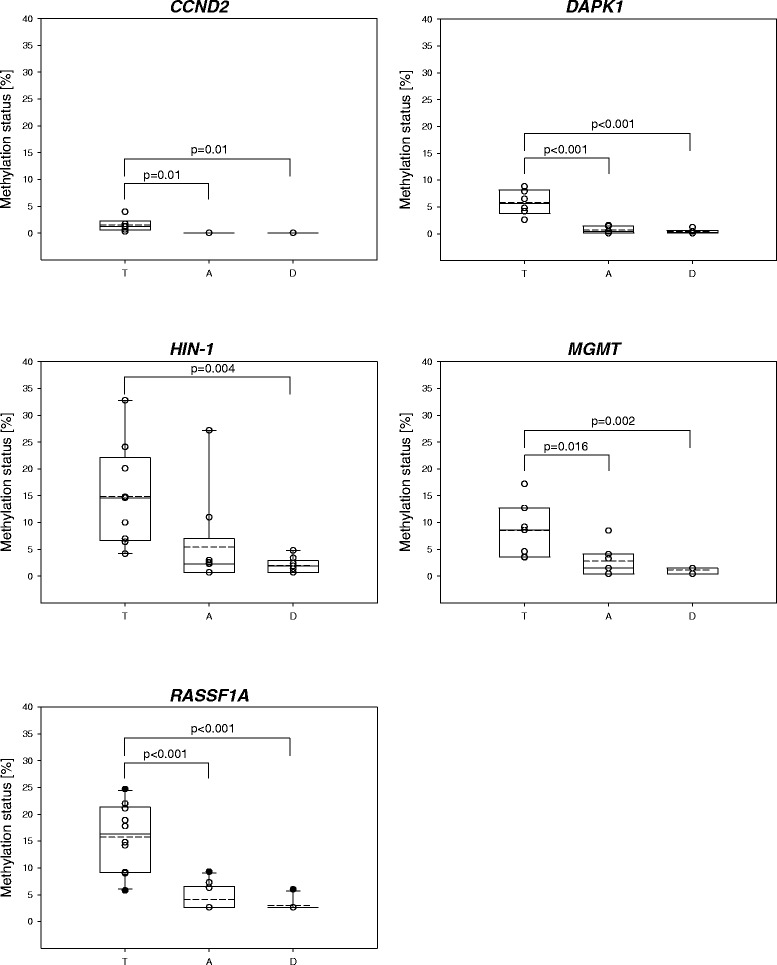
Distribution of the methylation status in tumor (*T*), tumor-adjacent (*A*) and tumor-distant (*D*) tissues. Patients have only been included if the methylation status in the tumor tissue was ≥ LOQ. *Straight line*: median, *dashed line*: arithmetic mean. *LOQ* limit of quantification

Figure 4 illustrates the distribution of the promoter methylation levels in tumor, tumor-adjacent and tumor-distant tissues as well as in normal tissues of the control group. In order to be able to include methylation levels < LOD and < LOQ, values < LOD and < LOQ were substituted with default values, namely half the LOD and half the LOQ of the certain MS-HRM method, respectively. Figure 5 is limited to patients showing methylation status ≥ LOQ in the tumor tissue. As already discussed above, methylation levels were generally very low. In none of the samples did any of the genes show methylation status > 33 %. In only 18 % (3/17) of the tumor tissues, the methylation status of *HIN-1* and *RASSF1A* was > 20 %. The methylation status of *CCND2* and *DAPK1* was generally < 10 %. In five tumor tissues, none of the six genes showed a methylation status ≥ LOQ.

Statistically significant differences between the DNA methylation status in tumors and normal breast tissues of the control group were found for *DAPK1* (*p* = 0.012*)*, *HIN-1* (*p* = 0.005) and *RASSF1A* (*p* < 0.001). Figure 4 indicates that in the case of *MGMT*, tumors showed a higher methylation status than normal breast tissues of the control group. Since in one of four women of the control group *MGMT* was methylated (methylation status 5.1 %), the difference was, however, not found to be statistically significant.

**Fig. 6:**
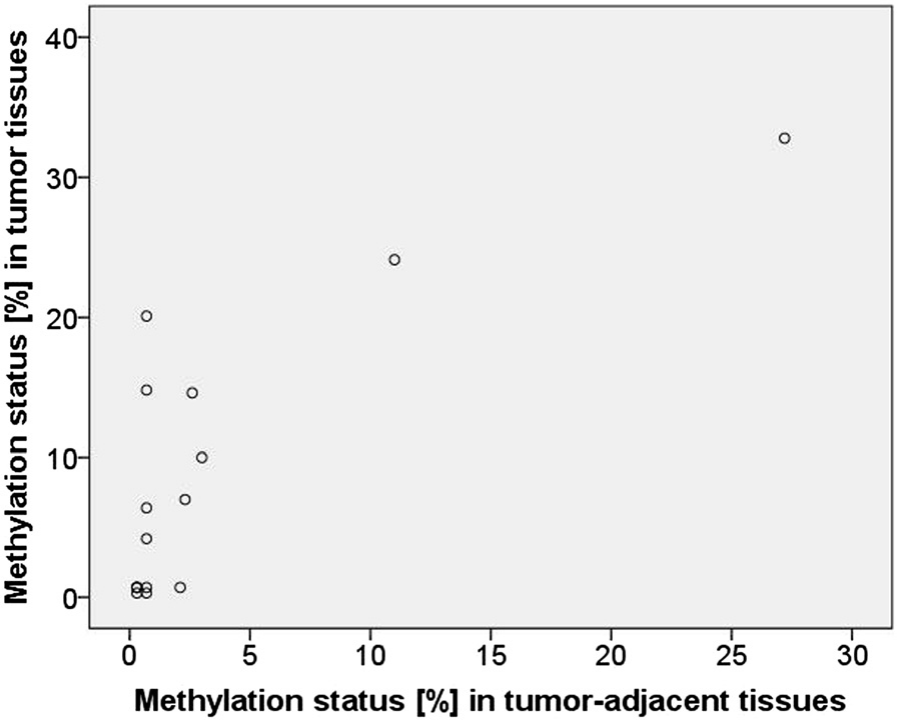
Correlation of the methylation status of the *HIN-1* promoter in tumor and tumor-adjacent tissues

For all genes except *GSTP1*, statistically significant differences were found between the methylation status in tumor and tumor-distant tissues of breast cancer patients. This holds for all patients (Fig. 4) and the subgroup (methylation status of the certain gene promoter in the tumor ≥ LOQ, Fig. 5). The methylation status of *RASSF1A*, *DAPK1* and *CCND2* in tumor-adjacent tissues was significantly different from that in tumors. A statistically significant difference between the methylation status in tumor and tumor-adjacent tissues was also found for *MGMT,* but only for the subgroup (Fig. 5). In case of *HIN-1*, the methylation status in tumor-adjacent tissues strongly correlated with that in tumors (r = 0.785, *p* < 0.001) (Fig. 6), but not with that in tumor-distant tissues (r = 0.312, *p* = 0.239). For *HIN-1*, *RASSF1A* and *MGMT*, the methylation status in tumor-adjacent tissues was higher than that in tumor-distant tissues but the differences were not statistically significant.

### Association between methylation status and clinicopathological parameters

When we investigated if the methylation status of the six tumor suppressor gene promoters is associated with clinicopathological characteristics (Table 1) of the breast cancer patients, we used the methylation status as categorical variable and divided the data set into two categories: “methylation status < LOD” and “methylation status > LOD”.

**Fig. 7:**
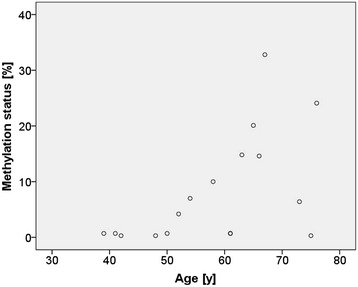
Correlation of *HIN-1* promoter methylation in tumor tissues with the age of the patients

The methylation status of *HIN-1* in tumor tissues correlated with the age of the patients (r = 0.555, *p* = 0.021) (Fig. 7). In addition, we found association between the methylation status of *HIN-1* in the adjacent tissues and the HER2 status (*p* = 0.039). In patients with HER2-positive tumors, the methylation status of *HIN-1* was more frequently < LOD than in patients with HER2-negative status. The methylation status of *MGMT* in tumor-distant tissues was associated with tumor grading (*p* = 0.019). In patients with tumor grade 2, the methylation status of *MGMT* in tumor-distant tissues was most frequently < LOD whereas in patients with tumor grade 3, the *MGMT* promoter was found to be methylated.

### Correlation between the methylation status of different genes

**Fig. 8:**
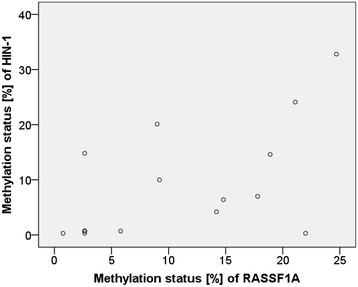
Correlation of the methylation status of *HIN-1* promoter and *RASSF1A* promoter in tumor tissues

We also evaluated if there is a statistically significant correlation between the promoter methylation status of different tumor suppressor genes in tumor tissues of one and the same patient. Correlation analyses revealed that the methylation status of *RASSF1A* positively correlated with that of *HIN-1* (r = 0.600, *p* = 0.011) (Fig. 8) and *MGMT* (r = 0.523, *p* = 0.031). In addition, we found a positive correlation between promoter methylation of *DAPK1* and *MGMT* (r = 0.514, *p* = 0.042).

## Discussion

We determined the methylation status of a panel of six tumor suppressor gene promoters in tumor, tumor-adjacent and tumor-distant tissues of 17 breast cancer patients. *CCND2*, *DAPK1*, *GSTP1*, *HIN-1*, *MGMT* and *RASSF1A* were selected because they have previously been reported to be frequently methylated in primary breast cancers. Tumor-adjacent and tumor-distant tissues appeared histologically normal and were located about 1 cm and about 3 cm from the tumor, respectively. Normal breast tissues of women that had been undergoing reduction mammoplasty served as control.

The DNA methylation status was determined by MS-HRM analysis, which consists of the following steps: treatment of the DNA with sodium bisulfite in order to convert unmethylated cytosines into uraciles (methylated cytosines remain unchanged); amplification of the bisulfite-treated DNA by PCR (uracils are replicated as thymines and methylated cytosines as cytosines); brief denaturation and rapid reannealing of the PCR products; and finally high-resolution melting by gradually increasing the temperature. Guanine–cytosine-rich sequences (resulting from methylated CpG dinucleotides) melt at higher temperature than adenine–thymine-rich sequences (resulting from unmethylated CpG dinucleotides). Melting curves are obtained by plotting the fluorescence intensity against the temperature. The methylation status of unknown samples can be determined with the help of a calibration curve established by analyzing mixtures of unmethylated and fully methylated control DNA.

In our study MS-HRM analysis was the method of choice because we aimed at determining very low methylation levels and discriminating between small differences in the methylation status of the tumor suppressor gene promoters. MS-HRM methods allow adjusting the accessible methylation range by generating a (helpful) bias toward methylated or unmethylated alleles in PCR amplification [29]. High sensitivity can be achieved by using primers containing a low number of CpG dinucleotides, favoring amplification of guanine–cytosine-rich sequences (resulting from methylated CpG dinucleotides) [23]. In addition, the T_a_ and the MgCl_2_ concentration can be varied in order to tailor the methylation range accessible. LODs (S/N = 3) and LOQs (S/N = 10) of the MS-HRM methods applied were determined by repeatedly analyzing bisulfite-treated, unmethylated control DNA. The methods proved to be very sensitive, with LODs and LOQs ranging from 0.1 to 1.5 % and 0.3 to 5.3 %, respectively.

In contrast to several other methods used in DNA methylation analysis, e.g., bisulfite pyrosequencing, MS-HRM methods do not allow determining the methylation status of individual CpG dinucleotides. From the melting profiles and the corresponding derivative plots one can, however, assess if the original template was methylated homogeneously or heterogeneously. In the reannealing step, carried out after briefly denaturing the PCR products, DNA sequences originating from homogenously methylated templates form homoduplexes, whereas those originating from partially methylated templates form heteroduplexes. Due to base-pairing mismatches, heteroduplexes begin to melt at lower temperature and lead to more complex melting profiles than homoduplexes. In biopsy samples analyzed in the present study, promoters of *CCND2*, *GSTP1*, *HIN-1* and *RASSF1A* were found to be methylated homogenously (in case they were methylated at all). Most of the tumors showed homogenous methylation of *DAPK1* and *MGMT*, in some tumor tissues the promoter of these genes was methylated heterogeneously. We did not find an association between the occurrence of heterogeneous methylation in *DAPK1* and *MGMT* promoters and any of the clinicopathological parameters of the patients. Heterogeneous methylation of *DAPK1* promoter has been shown previously in patients with chronic lymphocytic leukemia [30] or diffuse large B-cell lymphoma [31]. The *MGMT* promoter has been found to be methylated heterogeneously in patients with breast cancer [24], in diffuse large B-cell lymphoma [31] and in human breast cancer cell line HS578T [32].

In MS-HRM analysis, the DNA methylation status is determined with the help of calibration curves established by analyzing mixtures of unmethylated and methylated human control DNA. In case of heterogeneous methylation, melting curves differ in shape from melting curves obtained for standards. In order to obtain accurate results in spite of differences in the shape of the melting curves, we established interpolation calibration curves by taking into account the average of the normalized fluorescence signal for each standard over the entire temperature.

None of the normal breast tissues of the control group showed methylation of *DAPK1*, *HIN-1* and *RASSF1A*. In one tissue, the methylation status of *GSTP1* was < LOQ. In the breast tissue of the oldest woman (age: 60 years), the promoters of *CCND2* and *MGMT* were found to be methylated (*CCND2* < LOQ, *MGMT*: 5.2 %). However, for none of the tumor suppressor genes did we find an association between the age of the women and the DNA methylation status.

In breast cancer patients, 94 % and 82 % of the tumor tissues showed methylation of *RASSF1A* and *HIN-1* promoters, respectively. In 82 % of the tumors, even both promoters were methylated. Our results are in accordance with previous studies reporting frequent methylation of *RASSF1A* and/or *HIN-1* in breast carcinoma. *RASSF1A* methylation was found in 85 % [33–35], 68 % [36], 65 % [37], 59 % [38], 58 % [21] and 33 % [24] of the breast tumors analyzed, *HIN-1* in 74 % [39]), 73 % [33] and 49 % [21] of the cases. In the present study, promoters of *MGMT*, *DAPK1* and *GSTP1* were methylated in 65 %, 63 % and 53 % of the tumors. However, in case of *GSTP1*, all tumors showed methylation status < LOQ. Frequency of methylation of these promoters was higher than reported in the literature (*MGMT*: 22 % [24]; *DAPK1*: 50 % [37] and 37.5 % [24]; *GSTP1*: 16.6 % [24] and 14 % [40]), most probably due to the high sensitivity (LOD < 1 %) of the MS-HRM methods applied in the present study. In contrast, the *CCND2* promoter was found to be less frequently methylated (in 35 % of the tumors) than in the study of Lewis et al*.* (57 % [38]).

In each tumor tissue, the promoter of at least one of the six genes was found to be methylated. A total of 71 % of the tumors showed promoter methylation of ≥ 4 genes. This finding is consistent with previous studies reporting methylation of more than one tumor suppressor gene in breast tumors [24, 37]. Simultaneous methylation of several tumor suppressor genes indicates the important role DNA methylation is playing in breast cancer development.

In tumors, the DNA methylation status of *DAPK1* (*p* = 0.012*)*, *HIN-1* (*p* = 0.005) and *RASSF1A* (*p* < 0.001) was statistically significantly different from that in normal breast tissues of the control group. In addition, tumor tissues showed a higher methylation status of *MGMT* (Fig. 4) compared to the control group, however, the difference was not statistically significant since one normal breast tissue had a methylation status of 5.1 % (other normal breast tissues of the control group had a methylation status < LOD). In the present study, *MGMT* promoter methylation was detected in the oldest woman of the control group. In a previous study, *MGMT* methylation has been associated with the age of breast cancer patients [24]. However, in our study we did not find a significant correlation between the DNA methylation status of *MGMT* and the age of the women, neither for breast cancer patients nor for the control group.

With the exception of *CCND2* and *DAPK1*, the genes were not only frequently methylated in the tumors but also in the corresponding tumor-adjacent and tumor-distant tissues of the breast cancer patients. The *RASSF1A* promoter was as frequently and the *HIN-1*, *MGMT* and *GSTP1* promoters were almost as frequently methylated as in tumors. With the exception of *GSTP1*, the methylation status was significantly lower in tumor-distant tissues than in tumors. In case of *RASSF1A*, *HIN-1* and *MGMT* promoters, tumor-adjacent tissues showed higher methylation status than tumor-distant tissues, the difference was, however, not statistically significant. These results demonstrate that the methylation status of *RASSF1A*, *HIN-1* and *MGMT* promoters indicates field cancerization in breast cancers. To the best of our knowledge, field cancerization due to *MGMT* promoter methylation has not been reported so far. *MGMT* field cancerization has, however, been detected in colorectal cancers [14, 41] and oral squamous cell carcinomas [42]. Aberrant methylation in tissue adjacent to breast tumor has already been reported previously for *RASSF1A* [20, 21] and *HIN-1* [21]. Methylation of the *HIN-1* promoter has been detected with high frequency (95 %) in preinvasive lesions such as ductal and lobular carcinoma in situ [39]*,* indicating that it is an early event in breast tumorigenesis. In the present study, the methylation status of the *HIN-1* promoter in tumor-adjacent tissues was found to correlate strongly with that in the corresponding tumors, but not with that in the corresponding tumor-distant tissues. Among the promoters investigated in the present study, the methylation status of the *HIN-1* promoter can thus be considered the best suitable biomarker for detecting field cancerization. Further investigation is needed to test whether it can be used for defining surgical margins in order to prevent future recurrence of breast cancer.

Breast cancer is known to be a heterogeneous disease with regard to histopathological and molecular characteristics, outcome and response to treatment. Previous studies have reported association between the methylation status of a variety of gene promoters and clinicopathological parameters.

*HIN-1* is a putative cytokine reported to be highly expressed in normal but not cancerous mammary epithelial cells [39, 43]. The methylation status of *HIN-1* in breast tumors has been associated with the ER, PR and/or HER2 status [21, 43]. In our study, in tumor-adjacent tissues of patients with HER2-positive tumors the *HIN-1* promoter was more frequently unmethylated (< LOD) than in those from patients with HER2-negative status. In addition, we found association between the methylation status of *HIN-1* in the tumor-distant tissue and the PR and ER status of the tumor. However, the number of PR-negative and ER-negative tumors investigated was too low to allow a statistical conclusion. The methylation status of the *HIN-1* promoter in tumors was found to correlate with the age of the patients, which is in accordance with the study of Feng et al*.* [21].

*MGMT* plays a role in DNA repair. Silencing of *MGMT* by promoter methylation is a predictor of overall survival and response to alkylating agents [44]. In our study, the methylation status of *MGMT* in tumor-distant tissues was associated with tumor grading. In patients with tumor grade 3, the *MGMT* promoter was found to be more frequently methylated than in patients with tumor grade 2. In the study of Tserga et al*.* [24], the methylation status of the *MGMT* promoter in the tumor itself was found to be associated with advanced tumor grade.

Several studies report association between *RASSF1A* promoter methylation and ER status of the tumor [35, 45, 46]. In the present study only one of the tumors was ER-negative. We therefore could not evaluate potential association between the methylation status of the *RASSF1A* promoter and the ER status of the tumors.

As already mentioned above, more than one tumor suppressor gene has been found to be methylated in a high percentage of the tumors. Correlation analyses revealed that the methylation status of *RASSF1A* positively correlated with that of *HIN-1* and *MGMT*. In addition, we found a statistically significant positive correlation between the promoter methylation of *DAPK1* und *MGMT*. Correlation between methylation levels of *RASSF1A* and *HIN-1* in tumor of breast cancer patients has been previously published by Feng et al*.* [21].

## Conclusions

MS-HRM analysis was carried out to determine the methylation status of *CCND2*, *DAPK1*, *GSTP1*, *HIN-1*, *MGMT* and *RASSF1A* promoters in tumor tissues and histologically normal-appearing tumor-adjacent and tumor-distant tissues of 17 breast cancer patients. More than half of the tumors showed promoter methylation of *RASSF1A*, *HIN-1*, *MGMT*, *DAPK1* and *GSTP1*. In a high percentage of the tumors, more than one gene promoter was methylated. The methylation status of the *HIN-1* promoter was found to correlate with the age of the patients. Not only the tumors but also the corresponding tumor-adjacent and tumor-distant tissues frequently showed DNA methylation. The methylation status of the *RASSF1A*, *HIN-1* and *MGMT* promoters was significantly lower in tumor-distant tissues than in tumors. In tumor-adjacent tissues, the methylation status of these gene promoters was found to be higher than in tumor-distant tissues, indicating field cancerization. So far, field cancerization due to *MGMT* promoter methylation has been reported for colorectal cancers and oral squamous cell carcinomas, but not for breast cancer. In the case of the *HIN-1* promoter, the methylation status in tumor-adjacent tissues correlated strongly with that in the corresponding tumor tissues. Among the promoters investigated, *HIN-1* promoter methylation can therefore be considered the best suitable biomarker for detecting field cancerization. Further studies should be carried out to investigate its applicability for defining surgical margins in order to prevent future recurrence of breast cancer.

## Abbreviations

CCND2: cyclin D2
CDH13: cadherin-13
CpG: cytosine-phosphatidyl-guanosine
DAPK1: death-associated protein kinase 1
ER: estrogen receptor
GSTP1: glutathione S-transferase pi 1
IDC: invasive ductal carcinoma
ILC: invasive lobular carcinoma
HER2: human epidermal growth factor receptor 2
HIN-1: high in normal 1
LOD: limit of detection
LOQ: limit of quantification
MGMT: O-6-methylguanine-DNA methyltransferase
MIB-1: mindbomb E3 ubiquitin protein ligase 1
MS-HRM: methylation-sensitive high-resolution melting
PBS: phosphate-buffered saline
PCR: polymerase chain reaction
PR: progesterone receptor
RASSF1A: Ras association domain family member 1
RIL: reversion-induced LIM protein
S/N: signal-to-noise ratio
T_a_: annealing temperature
